# CCR5, CD163, CSF1R, and FCGR3A as Candidate Biomarkers Associated With Mitophagy in Atherosclerosis

**DOI:** 10.1155/humu/3810886

**Published:** 2026-10-22

**Authors:** Jiadong Fu, Yongda Dong

**Affiliations:** ^1^ Department of Cardiology First Hospital of Quanzhou Affiliated to Fujian Medical University Quanzhou City China fjmu.edu.cn

**Keywords:** atherosclerosis, mitophagy, molecular docking, scRNA-seq, WGCNA

## Abstract

Atherosclerosis is a chronic inflammatory disease, and the role of mitophagy in its pathogenesis remains unclear. To address this, this study integrated atherosclerosis‐related transcriptome data and single‐cell sequencing data from the GEO database, obtained mitophagy‐related genes from MSigDB, and assessed mitophagy activity via GSEA and ssGSEA; WGCNA was used to screen disease‐ and mitophagy‐associated module genes, and their intersection with differentially expressed genes yielded 286 candidate genes. A protein–protein interaction (PPI) network and cytoHubba algorithms were subsequently used to prioritize key genes, and dual machine learning algorithms (LASSO regression and SVM‐RFE) to screen potential biomarkers, which were designated as CCR5, CD163, CSF1R, and FCGR3A. The expression patterns of all four candidates were further assessed in the independent GSE43292 dataset and were concordant with those observed in the discovery dataset. GSEA of samples stratified by candidate‐gene expression identified enrichment of gene sets related to myogenesis, allograft rejection, and inflammatory responses; candidate drug‐target associations were predicted using DSigDB, and exploratory AutoDock analysis suggested a feasible interaction between sunitinib and CSF1R. Additionally, exploratory single‐cell analysis of three atherosclerosis patients identified seven major cell populations, with CD163, CSF1R, and FCGR3A showing macrophage‐enriched expression within this dataset. Collectively, this study provides hypothesis‐generating evidence for an association between mitophagy‐related transcriptional patterns and atherosclerosis. Four candidate biomarkers (CCR5, CD163, CSF1R, and FCGR3A) were prioritized, and a potential CSF1R‐sunitinib interaction was proposed for future experimental validation.

## 1. Introduction

Atherosclerosis is a chronic, progressive disease that serves as the primary pathological basis for various cardiovascular and cerebrovascular disorders [1, 2]. Its clinical manifestations include ischemic peripheral artery disease, ischemic heart disease, and ischemic stroke [3, 4]. The primary pathological feature of atherosclerosis is the formation of atherosclerotic plaques within the vascular endothelium, predominantly affecting large and medium‐sized arteries [5]. As the disease progresses, vascular wall hardening and lumen narrowing occur, ultimately triggering severe cardiovascular events [6]. Dyslipidemia represents the core pathogenesis of atherosclerosis, making lipid management—primarily focused on lowering low‐density lipoprotein cholesterol—a key strategy for its prevention and control [7, 8]. Statistics indicate that approximately 20 million people worldwide die annually from atherosclerotic diseases, making it a serious threat to human health and life [9]. However, the underlying mechanisms of atherosclerosis remain incompletely understood. Therefore, in‐depth research into the pathogenesis of atherosclerosis and the identification of novel prognostic and therapeutic targets are imperative.

Mitophagy is the selective engulfment and degradation of damaged mitochondria by cells through autophagy mechanisms, thereby maintaining mitochondrial and cellular homeostasis [10]. Generally, mitophagy activation depends on two pathways: the ubiquitin‐dependent pathway and the ubiquitin‐independent pathway. The ubiquitin‐dependent pathway relies on extensive ubiquitination of mitochondrial surface proteins to promote mitophagy, including the PINK1/Parkin pathway. In this pathway, loss of mitochondrial membrane potential impairs the entry of PINK1 into the inner mitochondrial membrane, causing PINK1 to accumulate stably on the cytoplasmic face of the outer mitochondrial membrane [11, 12]. Concurrently, PINK1 recruits and activates Parkin, an RBR‐family E3 ubiquitin ligase, by relieving its autoinhibited conformation. Activated Parkin then ubiquitinates mitochondrial outer membrane proteins, thereby promoting mitophagy and mitochondrial quality control [13]. Appropriate mitophagy plays a vital role in protecting vascular endothelial cells, inhibiting foam cell formation, and delaying atherosclerosis. Studies indicate that mitochondrial dysfunction can disrupt smooth muscle cell proliferation, endothelial cell function and survival, and macrophage polarization through mitochondrial oxidative stress, imbalanced mitochondrial dynamics, and impaired autophagy, thereby promoting the development of atherosclerotic lesions [14]. Endothelial cells serve as a critical barrier protecting the vascular wall against stimuli, and endothelial injury represents the initiating event in atherosclerosis [15]. High glucose induces mitochondrial fragmentation in vascular endothelial cells, reduces expression of the autophagy marker LC3 II, and increases expression of Bcl‐2–associated proteins and cytochrome C, leading to endothelial injury. In contrast, mesenchymal stem cells activate the PINK1/Parkin mitophagy signaling pathway, accelerating the clearance of damaged mitochondria, inhibiting reactive oxygen species production, and mitigating cellular injury and apoptosis [16]. Therefore, elucidating the regulatory mechanisms of mitophagy during atherosclerosis progression holds significant value for identifying novel intervention targets and advancing cardiovascular disease prevention and treatment strategies.

The present study focused on the intersection between mitophagy‐related transcriptional activity and atherosclerosis. Pathway scoring, WGCNA, differential expression analysis, protein–protein interaction (PPI) network analysis, and two complementary machine learning algorithms were integrated to prioritize candidate biomarkers. Their cell‐type distribution was subsequently examined. Rather than proposing a new algorithmic framework, the distinctive contribution of this study lies in integrating mitophagy‐related transcriptional activity and macrophage‐enriched candidate biomarkers within a single analytical workflow. Drug prediction and representative molecular docking were used only as exploratory analyses to generate a testable CSF1R‐sunitinib hypothesis.

## 2. Materials and Methods

### 2.1. Data Sources and Processing

This study downloaded the atherosclerosis discovery dataset GSE100927, generated on the GPL17077 platform, from the Gene Expression Omnibus (GEO) database (https://www.ncbi.nlm.nih.gov/geo/), obtaining peripheral arterial tissue samples from 69 patients with atherosclerosis and 35 controls. Probe identifiers were mapped to gene symbols using the GPL17077 platform annotation file, and the expression values of multiple probes mapped to the same gene were averaged. The independent validation dataset GSE43292, generated on the GPL6244 platform, contained 32 carotid atherosclerotic plaque samples and 32 paired macroscopically intact arterial tissue samples obtained from the same patients [17]. GSE100927 and GSE43292 were processed and analyzed separately and were not merged; therefore, no cross‐dataset batch‐effect correction was applied. The single‐cell dataset GSE155514 was also downloaded from GEO and comprised carotid intima single‐cell RNA sequencing (scRNA‐seq) data from three patients with atherosclerosis. Given the limited number of donors, the single‐cell analysis was considered exploratory and was used primarily to examine the cell‐type distribution of the candidate genes. The REACTOME_MITOPHAGY.v2025.1.Hs dataset was obtained from the MSigDB database (https://www.gsea-msigdb.org/gsea/login.jsp), yielding 47 mitophagy‐related genes.

### 2.2. Enrichment Score Determination

Gene set enrichment analysis (GSEA) was performed using GSEA software (Version 4.4.0). The REACTOME_MITOPHAGY.v2025.1.Hs gene set was used to evaluate mitophagy‐related enrichment between atherosclerosis and control samples. For candidate gene–associated pathway analysis, samples in GSE100927 were divided into high‐ and low‐expression groups according to the median expression of CCR5, CD163, CSF1R, or FCGR3A, and GSEA was performed using the h.all.v2024.1.Hs.symbols Hallmark gene set collection. Per‐sample mitophagy‐related enrichment scores were calculated using the ssGSEA algorithm implemented in the GSVA R package (Version 1.50.5) [18].

### 2.3. WGCNA

This study screened target modules associated with mitophagy using the WGCNA R package (Version 1.73) [19]. A soft‐thresholding power of 3, at which the scale‐free topology fit index exceeded 0.90, was selected, with a minimum module size of 50 genes and a module merging height of 0.2. Modules showing an absolute correlation coefficient ≥ 0.5 with both the mitophagy‐related score and atherosclerosis status (*p* < 0.01) were selected as candidate modules. The gene significance (GS) and module membership (MM) values of genes were calculated, and genes with |GS| ≥ 0.4 and |MM| ≥ 0.8 were identified as hub genes.

### 2.4. Differential Expression Analysis

This study employed the limma R package (Version 3.58.1) [20] to analyze gene expression level differences between atherosclerotic and control samples. Significant differentially expressed genes (DEGs) were identified using the criteria |log_2_FC| ≥ 1 and adjusted *p* value < 0.01.

### 2.5. PPI Networks and Key Gene Screening

The 286 genes obtained from the intersection of the WGCNA hub genes and DEGs were submitted to the STRING database (https://cn.string-db.org/) to construct a PPI network. The resulting interaction network was imported into Cytoscape software (Version 3.10.3). Six algorithms implemented in the cytoHubba plugin (Version 0.1)—closeness, degree, edge percolated component (EPC), maximal clique centrality (MCC), maximum neighborhood component (MNC), and radiality—were applied. The Top 20 genes ranked by each algorithm were retained, and their intersection was used for subsequent analysis.

### 2.6. Machine Learning Algorithms for Candidate Biomarker Prioritization

This study applied LASSO regression and SVM‐RFE to prioritize candidate biomarkers for atherosclerosis. LASSO regression was performed using the glmnet R package (Version 4.1‐8), and the penalty parameter corresponding to the minimum cross‐validation error was selected. SVM‐RFE was performed using the e1071 R package (Version 1.7‐14), with recursive feature elimination used to determine the feature subset yielding the minimum cross‐validation error. Genes retained by both approaches were defined as candidate biomarkers.

### 2.7. External Validation of Candidate‐Biomarker Expression

The independent GSE43292 dataset was used to evaluate the reproducibility of the expression patterns of CCR5, CD163, CSF1R, and FCGR3A. This dataset included 32 carotid atherosclerotic plaque samples and 32 paired macroscopically intact arterial tissue samples from the same patients. Because the samples were paired within individuals, differences in gene expression between the two tissue types were evaluated using the paired Wilcoxon signed‐rank test.

### 2.8. Targeted Drug Prediction and Exploratory Molecular Docking

Drug enrichment analysis was performed using the enrichR R package (Version 3.2) and the DSigDB database. Because sunitinib‐related DSigDB entries were linked to CSF1R, the CSF1R‐sunitinib pair was selected as a representative drug‐target pair for exploratory molecular docking. The crystal structure of the CSF1R kinase domain (PDB ID: 8JOT) was obtained from the RCSB Protein Data Bank (https://www.rcsb.org/), and the three‐dimensional structure of sunitinib was downloaded from PubChem (https://pubchem.ncbi.nlm.nih.gov/). PyMOL Version 2.5.5 was used to remove water molecules, hydrogenate the receptor, and remove the original small‐molecule ligand. ChemBioOffice 2019 was used for ligand energy minimization. AutoDockTools Version 1.5.7 was subsequently used to perform molecular docking. A binding energy < −5 kcal/mol and an $\text{interaction}\;\text{distance}<3.5\;\overset{{}^{\circ}}{\text{A}}$ were considered indicative of a potentially favorable docking pose.

### 2.9. Single‐Cell RNA‐Sequencing Analysis

Single‐cell data were processed using the Seurat R package (Version 5.0.3). Cells with fewer than 200 or more than 4000 detected genes were excluded, leaving 7876 cells. Data were normalized using SCTransform (sctransform Version 0.4.1), followed by principal component analysis. Batch effects among samples were corrected using Harmony (Version 1.2.0). A K‐nearest‐neighbor graph was constructed using the FindNeighbors function based on the first 50 Harmony‐corrected dimensions (reduction =  ^“^harmony^”^), and UMAP was generated using the same Harmony embedding. Cells were clustered using the FindClusters function with a resolution of 0.1. Cell types were annotated according to known marker genes from the CellMarker database. Cluster‐specific marker genes were identified using FindAllMarkers with logfc.threshold = 0.25, min.pct = 0.30, and only.pos = TRUE.

### 2.10. Statistical Analysis

Unless otherwise specified, all computational analyses were performed using R software (Version 4.3.0). Differences between two independent groups were assessed using the Wilcoxon rank‐sum test, whereas the paired tissue samples in GSE43292 were compared using the Wilcoxon signed‐rank test. Correlations were evaluated using Spearman’s rank correlation test. Two‐sided *p* values < 0.05 were considered statistically significant.

## 3. Results

### 3.1. Mitophagy‐Related Transcriptional Activity Is Associated With Atherosclerosis

To examine the association between mitophagy‐related transcriptional activity and atherosclerosis, disease and control samples were compared using GSEA. GSEA showed significant differential enrichment of the REACTOME_MITOPHAGY gene set between the two groups (*p* < 0.05; Figure 1A). Per‐sample scores calculated using the mitophagy‐related gene set also differed significantly between the disease and control groups (Figure 1B).

**Figure 1:**
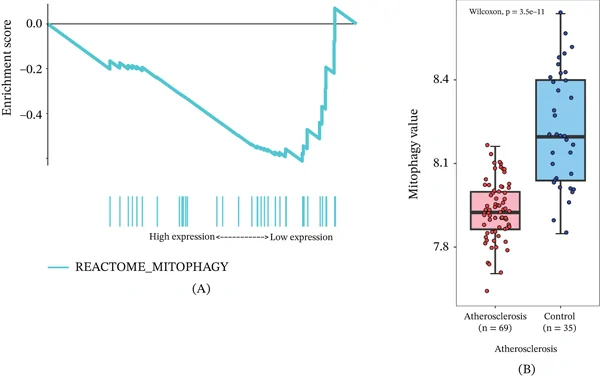
Association between mitophagy‐related transcriptional activity and atherosclerosis. (A) Enrichment results of mitophagy‐related pathways. (B) Relative levels of mitophagy scores between atherosclerosis and control groups.

### 3.2. WGCNA Analysis of Atherosclerosis

WGCNA analysis of the dataset revealed no obvious outlier samples (Figure 2A). A soft‐thresholding power of 3, at which the scale‐free topology fit index exceeded 0.90, was used to construct the coexpression network (Figure 2B). After calculating module–trait correlations, MEblue, MEbrown, and MEturquoise were identified as modules strongly correlated with both atherosclerosis and mitophagy‐related scores. Hub genes were selected in these three modules based on |GS| ≥ 0.4 and |MM| ≥ 0.8 (Figure 2C).

**Figure 2:**
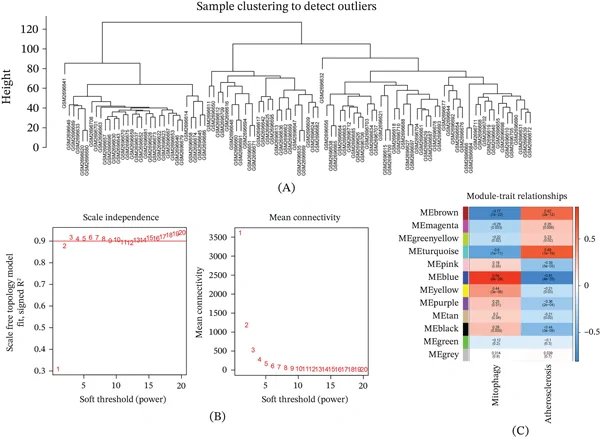
WGCNA screening for mitophagy‐related genes. (A) Sample clustering tree of atherosclerosis samples. (B) Soft thresholding selection plot. A soft‐thresholding power of 3, at which the scale‐free topology fit index exceeded 0.90, was selected to construct the coexpression network. (C) Module–trait correlation plot. The numbers within squares denote correlation coefficients. Numbers in parentheses indicate significance *p* values. The *y*‐axis and *x*‐axis represent modules and traits. Red indicates the positive correlation, and blue indicates negative correlation.

### 3.3. Screening of Genes Related to Atherosclerosis and Mitophagy

Differential expression analysis between atherosclerosis and control groups identified 429 DEGs, comprising 315 upregulated genes and 114 downregulated genes (Figure 3A). The 20 most upregulated and 20 most downregulated DEGs were visualized in an expression heatmap (Figure 3B). We intersected hub genes from the mitophagy‐ and atherosclerosis‐associated WGCNA modules with the DEGs, yielding 286 candidate genes (Figure 3C).

**Figure 3:**
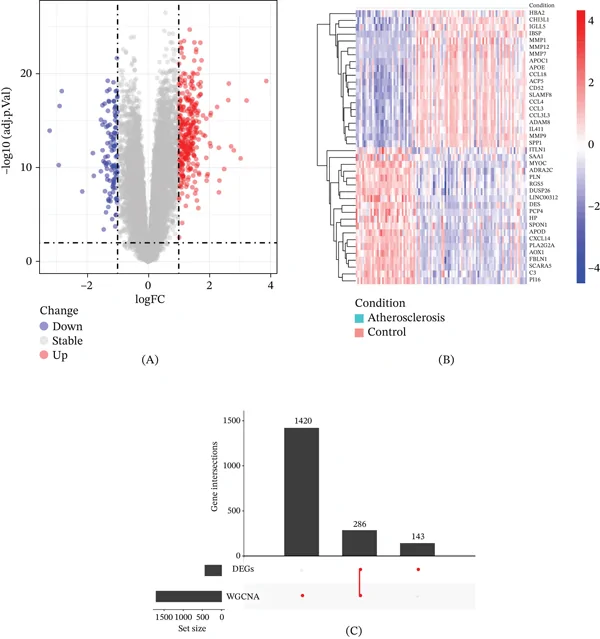
Differential expression analysis of atherosclerosis samples. (A) Volcano plot of DEGs in atherosclerosis, with log_2_FC on the *x*‐axis and −log_10_ (adjusted *p* value) on the *y*‐axis. (B) Heatmap of the 20 most upregulated and 20 most downregulated DEGs, where red indicates high expression, and blue indicates low expression. (C) Upset plot showing the intersection between WGCNA and DEGs.

### 3.4. Construction of the Protein Interaction Network for Atherosclerosis‐Related Molecules

We constructed a PPI network using the 286 genes obtained from the intersection of the WGCNA hub genes and DEGs as input to the STRING database (Figure 4A). The resulting PPIs were imported into Cytoscape software. Using the six algorithms implemented in the cytoHubba plugin, the Top 20 genes ranked by each algorithm were selected. The intersection of these 6 sets yielded 14 candidate genes (Figure 4B).

**Figure 4:**
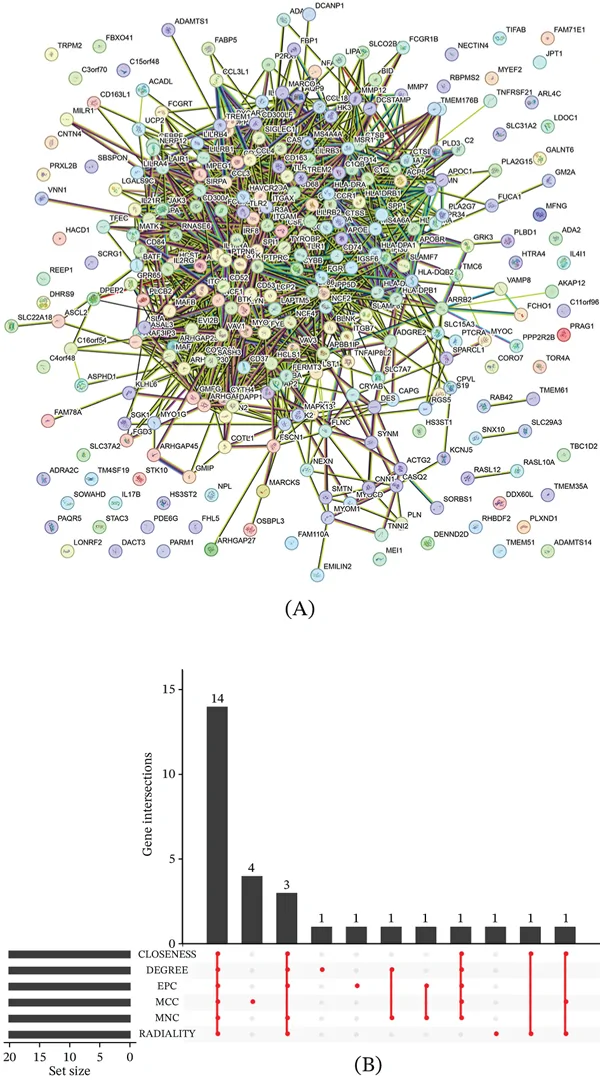
Construction of the PPI network. (A) PPI network constructed using the 286 genes obtained from the intersection of the WGCNA hub genes and DEGs as input to STRING, where each node represents a protein encoded by the corresponding gene. (B) Upset plot showing the intersection of the 14 genes identified by six cytoHubba algorithms.

### 3.5. Machine Learning–Based Prioritization of Candidate Biomarkers

LASSO regression was applied to the 14 candidate genes, and the penalty parameter corresponding to the minimum cross‐validation error was lambda.min = 0.0273 (Figure 5A,B). SVM‐RFE was applied to the same gene set, with the minimum cross‐validation error observed at 13 variables (*N* = 13; Figure 5C). The intersection of the LASSO and SVM‐RFE feature sets yielded four candidate biomarkers: CCR5, CD163, CSF1R, and FCGR3A (Figure 5D).

**Figure 5:**
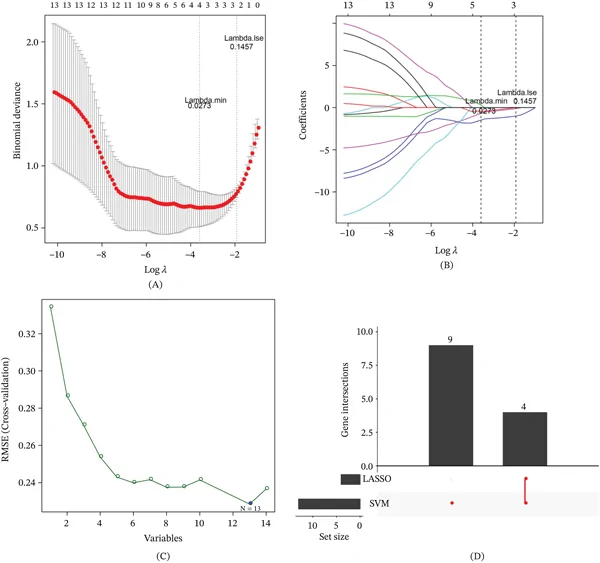
Machine learning–based prioritization of candidate biomarkers. (A) LASSO penalty parameter plot, with log(lambda) on the *x*‐axis and degrees of freedom on the *y*‐axis. (B) LASSO regression coefficient plot, with log(*λ*) on the *x*‐axis and gene coefficients on the *y*‐axis, illustrating how coefficients change across different variables after *λ* penalty. (C) Relationship plot between SVM‐RFE cross‐validation error and feature count. *x*‐axis: number of genes in each iterative model. *y*‐axis: root mean square error. (D) Upset plot showing the intersection of key genes identified by LASSO and SVM‐RFE.

### 3.6. External Validation of Candidate‐Biomarker Expression

The expression patterns of the four candidate biomarkers were further evaluated in the independent GSE43292 dataset. CCR5 (*p* = 0.00034), CD163 (*p* = 5.5 × 10^−7^), CSF1R (*p* = 2.3 × 10^−7^), and FCGR3A (*p* = 0.00036) showed significantly higher expression in atherosclerotic plaque tissues than in paired macroscopically intact arterial tissues. These expression patterns were concordant with those observed in the discovery dataset, providing independent support for the reproducibility of the four candidates (Figure S1A, S1B, S1C, S1D).

### 3.7. GSEA of Pathways Associated With Candidate Biomarker Expression

To explore pathways associated with candidate biomarker expression, samples were divided into high‐ and low‐expression groups according to the median expression of each gene, followed by GSEA. MYOGENESIS, ALLOGRAFT_REJECTION, and INFLAMMATORY_RESPONSE were among the gene sets enriched in comparisons based on the expression of all four candidate genes (Figure 6A–D). These results indicate pathway‐level associations and do not establish functional roles for the candidate genes.

**Figure 6:**
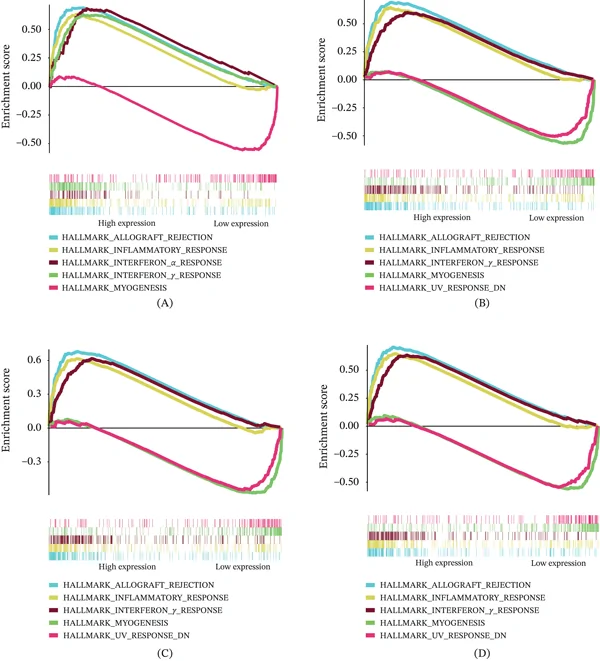
GSEA of pathways associated with candidate biomarker expression. (A) GSEA enrichment analysis for high‐ and low‐expression groups of CCR5. (B) GSEA analysis results for CD163 in the two groups. (C) GSEA analysis results for CSF1R in the two groups. (D) GSEA analysis results for FCGR3A in the two groups.

### 3.8. Prediction of Candidate Compounds and Exploratory Molecular Docking

DSigDB analysis identified several candidate compounds associated with the four biomarkers. Sunitinib‐related entries were linked to CSF1R; therefore, this pair was selected for representative exploratory docking. Using the CSF1R structure 8JOT, the predicted binding energy was −5.54 kcal/mol. The docking pose showed interactions with THR654 and VAL659 at distances of 3.1 and 3.2 Å, respectively (Figure S2). These computational results suggest a potentially feasible interaction but do not constitute experimental target validation.

### 3.9. Exploratory Single‐Cell Characterization of Atherosclerotic Tissue

Exploratory analysis of the three available atherosclerosis samples identified seven major cell populations following cell filtering, normalization, batch effect correction, dimensionality reduction, and clustering. Specifically, smooth muscle cells were annotated using MYH11, RGS5, MYL9, and ACTA2; macrophages using HLA‐DQA1, C1QA, C1QC, and C1QB; endothelial cells using VWF, PECAM1, EGFL7, and PTPRB; T cells using CD2, CD3D, CD3E, and GZMA; endothelial cell_2 using PLVAP, RAMP2, EMCN, and VWA1; mast cells using CPA3, MS4A2, KIT, and TPSAB1; and B cells using CD79A and IGKC (Figure S3A, S3B, S3C, S3D). Within this dataset, FCGR3A, CSF1R, and CD163 showed comparatively high expression in macrophages (Figure S4A, S4B, S4C); these observations require confirmation in larger, independent single‐cell cohorts.

## 4. Discussion

Atherosclerosis is a complex disease characterized by chronic inflammation, endothelial injury, lipid deposition, and immune‐cell activation [21, 22]. In this study, mitophagy‐related transcriptional activity differed between atherosclerotic and control tissues. By integrating pathway scoring, coexpression analysis, differential expression analysis, PPI network analysis, complementary machine learning methods, and exploratory single‐cell localization, four candidate biomarkers were prioritized, independently evaluated in GSE43292, and examined within their biological context. The contribution of this study lies in linking mitophagy‐related activity and macrophage‐enriched expression patterns, rather than in the novelty of the individual algorithms. These findings are associative and hypothesis‐generating and provide a basis for future diagnostic and mechanistic validation.

The four candidate genes prioritized in this study—CCR5, CD163, CSF1R, and FCGR3A—were associated with atherosclerosis and mitophagy‐related transcriptional patterns. Among these biomarkers, CCR5 is a G protein–coupled chemokine receptor that binds ligands including CCL3, CCL4, and CCL5 and participates in leukocyte trafficking and inflammatory signaling. CCR5‐mediated signaling has been implicated in cardiovascular disease. For example, CCR2/CCR5 signaling mediates interactions between macrophages and smooth muscle cells in pulmonary hypertension [23]. The CCL3‐CCR5 axis can induce aortic inflammation and further aneurysm formation, while also exacerbating cardiovascular disease progression by upregulating matrix metalloproteinases such as MMP‐9 [24]. CD163 is a single‐transmembrane glycoprotein belonging to the scavenger receptor cysteine‐rich superfamily, primarily expressed in the monocyte‐macrophage lineage, particularly in anti‐inflammatory M2 macrophages. CD163^+^ macrophages can induce the endothelium–mesenchymal transition process in atherosclerosis, promoting proinflammatory NF‐*κ*B signaling, which triggers intraplaque hemorrhage and worsens disease progression [25]. CD163^+^ macrophages may exert context‐dependent effects in atherosclerotic plaques. Sakamoto et al. [26] demonstrated that CD163^+^ macrophages restrain vascular calcification by activating NF‐*κ*B signaling in vascular smooth muscle cells and augmenting hyaluronan synthase and hyaluronan production; this anticalcific response may nevertheless be associated with the development of rupture‐prone, high‐risk plaque phenotypes. The proliferation, differentiation, and survival of mononuclear phagocytes depend on signaling through the macrophage colony‐stimulating factor receptor (CSF1R). Deficiency in this gene directly impacts the homeostasis of the macrophage population and monocytes, which is critically important for atherosclerosis development [27]. FCGR3A exhibits strong associations with cardiovascular disease. A comprehensive analysis confirmed altered immune cell infiltration levels in heart failure patients, revealing FCGR3A expression clusters highly enriched in monocytes that significantly impact heart failure [28]. Furthermore, elevated expression of FCGR3A and CD14 predicts increased monocyte infiltration, serving as a potential predictor for cardiovascular disease events [29]. Thus, these four candidates showed disease‐associated expression patterns that were independently supported in GSE43292 and warrant further investigation. However, the present analyses do not establish diagnostic or prognostic performance, causal effects, or roles in atherosclerosis progression.

Exploratory single‐cell analysis indicated a heterogeneous cellular composition in the three available atherosclerotic tissue samples, including smooth muscle cells, endothelial cells, macrophages, and lymphocytes. Macrophages are important immune effector cells that may contribute to plaque development through both lipid clearance and inflammatory signaling [30]. Within the present dataset, FCGR3A, CSF1R, and CD163 showed relatively high expression in macrophages. This pattern is biologically plausible because CD163 is commonly used as a macrophage marker, CSF1R participates in macrophage survival and proliferation, and FCGR3A is involved in immune‐complex responses [31–33]. However, these observations describe cell type–associated expression patterns and do not demonstrate that these genes regulate macrophage function or mitophagy. Larger single‐cell cohorts and functional studies are required to validate these findings.

This study has several limitations. First, the analyses were based on public transcriptomic datasets. Although the expression patterns of the four candidate genes were independently evaluated in GSE43292, this validation dataset included paired vascular tissues from only 32 patients, and the present analysis did not assess diagnostic accuracy, prognostic performance, or clinical outcomes. In particular, the scRNA‐seq dataset comprised only three atherosclerosis patients and did not include a healthy single‐cell control group, which limits the statistical power and generalizability of the cell type–specific observations. Therefore, the single‐cell findings should be considered exploratory. Second, the identified biomarkers were derived from statistical associations and computational prioritization and do not establish causal relationships. Third, the DSigDB prediction and CSF1R‐sunitinib docking represent an exploratory in silico assessment of one predicted drug‐target pair and do not demonstrate therapeutic efficacy or the equivalent druggability of all four biomarkers. Additional larger and prospectively characterized cohorts, together with functional experiments, are needed to validate the clinical relevance and biological roles of these candidate biomarkers.

## 5. Conclusion

In summary, integrative bioinformatics analyses prioritized CCR5, CD163, CSF1R, and FCGR3A as candidate biomarkers associated with mitophagy‐related transcriptional features in atherosclerosis. Their concordant expression patterns were supported in the independent GSE43292 dataset. Exploratory single‐cell analysis suggested macrophage‐enriched expression of CD163, CSF1R, and FCGR3A, whereas molecular docking generated a testable hypothesis regarding a potential CSF1R‐sunitinib interaction. These findings identify associations and candidate targets for future validation rather than establishing causal mechanisms or therapeutic efficacy.

**Nomenclature**
DEGs: differential expressed genes
GEO: Gene Expression Omnibus
GS: gene significance
LASSO: least absolute shrinkage and selection operator
MM: module membership
PINK1: PTEN‐induced kinase 1
PPI: protein–protein interaction
scRNA‐Seq: single‐cell RNA sequencing
SVM‐RFE: support vector machines‐recursive feature elimination
WGCNA: weighted gene coexpression network analysis

## Author Contributions

Jiadong Fu (first and corresponding author) designed the study, performed experiments, analyzed data, drafted the manuscript, and secured funding; Yongda Dong assisted with data collection and manuscript revision.

## Funding

No funding was received for this manuscript.

## Disclosure

All authors have read and approved the final version of the manuscript.

## Ethics Statement

Ethical approval was not required because this study analyzed only publicly available, deidentified datasets and involved no new recruitment of human participants or collection of biological specimens.

## Consent

The authors have nothing to report.

## Conflicts of Interest

The authors declare no conflicts of interest.

## Supporting Information

Additional supporting information can be found online in the Supporting Information section.

## Supporting information


**Supporting Information 1** Figure S1: External validation of the four candidate biomarkers in GSE43292. (A–D) Expression levels of (A) CCR5, (B) CD163, (C) CSF1R, and (D) FCGR3A in 32 carotid atherosclerotic plaque samples and 32 paired macroscopically intact arterial tissue samples. *p* values were calculated using the paired Wilcoxon signed‐rank test.

**Supporting Information 2** Figure S2: Exploratory molecular docking of the representative CSF1R‐sunitinib pair. The cyan represents amino acids; the blue molecule represents the receptor protein; the green molecule represents the drug small molecule. Hydrogen bonds between the receptor protein and the drug small molecule are indicated by yellow dashed lines, with the numbers above representing the bond lengths in angstroms (Å).

**Supporting Information 3** Figure S3: Single‐cell clustering of atherosclerosis. (A) Distribution of cells across the three samples after Harmony‐based batch‐effect correction. (B) UMAP visualization of cell type distribution. (C) Expression levels of cell marker genes. (D) Expression levels of four biomarker genes.

**Supporting Information 4** Figure S4: Relative expression of biomarkers across cell subpopulations. (A–C) Violin plots showing expression levels of biomarkers (A) CD163, (B) CSF1R, and (C) FCGR3A across cell subpopulations.

## Data Availability

The datasets analyzed in this study are publicly available in the Gene Expression Omnibus repository under Accession Numbers GSE100927 (https://www.ncbi.nlm.nih.gov/geo/query/acc.cgi?acc=GSE100927), GSE43292 (https://www.ncbi.nlm.nih.gov/geo/query/acc.cgi?acc=GSE43292), and GSE155514 (https://www.ncbi.nlm.nih.gov/geo/query/acc.cgi?acc=GSE155514).
